# Supplementary material for: Brain-like associative learning using a nanoscale non-volatile phase change synaptic device array
Source: Front Neurosci. 2014 Jul 22;8:205. doi: 10.3389/fnins.2014.00205 (PMC4106403; doi:10.3389/fnins.2014.00205)

Supplementary Figure. Measurement setup used in experiments. Probe card that directly probes pads on memory chip are connected to switch matrix. Setup is controlled by computer program.

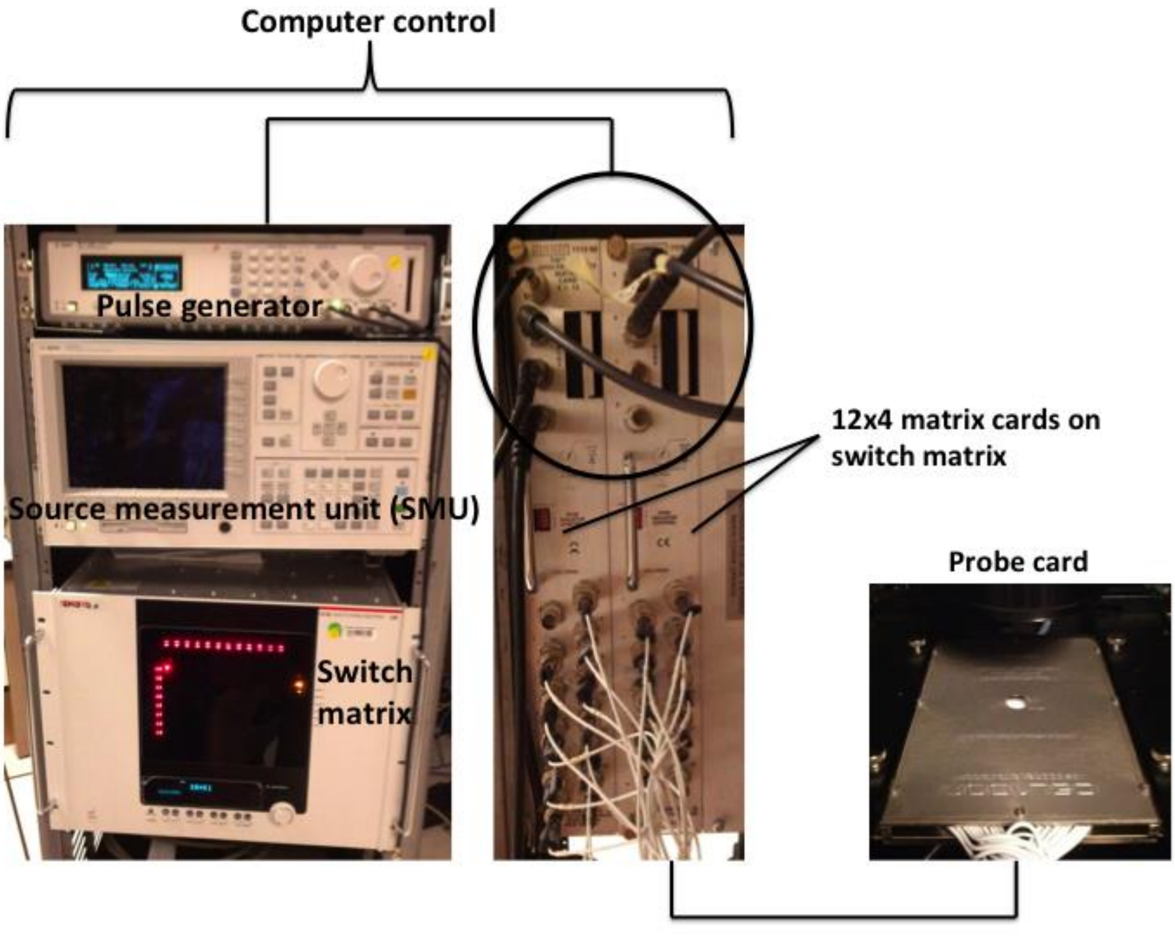

Supplement: Supplementary Figure 1 — Measurement setup used in experiments. Probe card that directly probes pads on memory chip are connected to switch matrix. Setup is controlled by computer program. [file Presentation1.PDF]
